# Hyperarousal Scale: Italian Cultural Validation, Age and Gender Differences in a Nonclinical Population

**DOI:** 10.3390/ijerph17041176

**Published:** 2020-02-12

**Authors:** Antonio Bruno, Amelia Rizzo, Maria Rosaria Anna Muscatello, Laura Celebre, Maria Catena Silvestri, Rocco Antonio Zoccali, Carmela Mento

**Affiliations:** 1 Department of Biomedical and Dental Sciences and Morphofunctional Imaging, University of Messina, Via Consolare Valeria 1, Contesse, 98125 Messina, Italy; mmuscatello@unime.it (M.R.A.M.); lallacelebre@gmail.com (L.C.); zoccali@unime.it (R.A.Z.); cmento@unime.it (C.M.); 2 Psychiatry Unit, Polyclinic Hospital University of Messina, Via Consolare Valeria 1, Contesse, 98125 Messina, Italy; amrizzo@unime.it (A.R.); mariacatenasilvestri89@gmail.com (M.C.S.)

**Keywords:** hyperarousal, H-Scale, validation, gender differences

## Abstract

Objectives. Studies on hyperarousal have increasingly developed in the last decade. Nevertheless, there are still very few valid measures of hyperarousal. The aim of the study is to verify the psychometric properties of the Italian version of the Hyperarousal Scale (H-Scale), in order to provide researchers with a valid measure for the target population. Method. The questionnaire was translated, back-translated, pre-tested, and cross-culturally adapted. Subsequently, the Italian version of the H-Scale, the Anxiety Sensitivity Index (ASI-3) and the Health Survey Questionnaire (SF-36) were administered to 982 adults, 456 males and 526 females, aged from 18 to 80 years (M = 35.61 ± 12.47). Results. Cronbach’s alpha of the translated H-Scale was 0.81. Furthermore, positive correlations with the ASI-3 and negative correlations with the SF-36 emerged. The H-Scale is also sensitive to catch age and gender differences. Conclusions. The Italian version of the H-Scale demonstrated good reliability and validity. Its sufficient discriminative and evaluative psychometric properties provide the theoretical evidence for further application in evidence-based research studies.

## 1. Introduction

Hyperarousal is an abnormal state of increased responsiveness to stimuli marked by various physiological and psychological symptoms, such as elevated heart rate and respiration and increased levels of alertness and anxiety. This state of amplified sensitivity is characterized by constant hypervigilance, difficulty in relaxing, increased anger or irritability, reckless or self-destructive behaviors, and increased startle response [1]. 

The DSM-5 resumes the hyperarousal state in Criterion E of post-traumatic stress disorder (PTSD), describing changes in the state of arousal, which started or worsened following the experience of a traumatic event, such as (a) irritability or aggressive behavior, (b) impulsive or self-destructive behavior, (c) feeling constantly “on guard” or like danger is lurking around every corner (or hypervigilance), (d) heightened startle response, (e) difficulty concentrating, and (f) sleep problems [2]. Research shows that among PTSD symptoms, hyperarousal is probably most closely related to impulsive behaviors; intense anxiety and discomfort associated with hyperarousal may lead subjects to look for immediate relief by acting impulsively, without considering possible outcomes. Furthermore, the experience of negative affective states characterized by high levels of arousal, along with emotion dysregulation, which has been found to be heightened among individuals with PTSD, are underlying mechanism of several specific impulsive behaviors, such as substance abuse, reckless driving, or drinking excessively [3].

Difficulty falling asleep is one of the core symptoms of hyperarousal. As a consequence, sleep deprivation can negatively affect mood, concentration, and the ability to cope with and manage stress, with potential long-term physical health consequences [4]. Given the major impact of hyperarousal on sleep quantity and quality, it is not surprising that its measurement appeared for the first time in clinical studies on insomnia and in research focused on neurological and neurophysiological aspects of hyperactivation. Regestein and colleagues [5] were the first to conduct an event-related potential (ERP) study in patients with insomnia, finding that the 26-item Hyperarousal Scale (H-Scale), empirically designed for measuring daytime alertness, accurately distinguished insomnia patients from controls. The insomnia group was characterized by higher alertness often accompanied by increased emotion, and furtherly confirmed by electrophysiological evidence of daytime arousal. Literature showed that the H-Scale scores were higher in insomniacs and correlated with the severity of insomnia [6] and that, in general, all sleep disorder groups had increased total hyperarousal scores. These self-report findings suggest that insomnia subjects may generally show more vulnerability to environmental requests [7]. 

In the past few years, it has become increasingly clear that hyperarousal can be a chronic condition interacting with many other psychiatric disorders, and that experiencing hyperarousal along with other symptoms can be tied to both physical and mental health. Hammad and colleagues [8] hypothesized a connection between hyperarousal and somatization, probably based on altered information processing; findings suggested that hyperarousal explained a substantial part of the variance (about 20%) of the construct of somatization.

Hyperarousal has also been related to depression. Szelenberger and Niemcewicz [9] found correlations between hyperarousal and depression scores, demonstrating relationships with the cognitive and affective aspects of depression; moreover, depressive symptoms and night eating were key factors related to insomnia [10]. Higher levels of self-reported arousal were associated with distress from interpersonal problems; individuals with insomnia who reported more distress from interpersonal problems tended to have more severe insomnia and cognitive pre-sleep arousal, possibly due to rumination [11].

Since women are more likely than men to suffer from PTSD [12] and major depression (MD) [13], a possible moderating role of hyperarousal on vulnerability to such disorders has been taken into account in order to explain gender differences in stress-related disorders. Preclinical data reported sex differences in stress response systems, as documented by differences in brain arousal centers and cellular and molecular mechanisms, such as receptor trafficking, cell signaling, hormone release, and peptide expression, highlighting sex differences that can be linked to increased arousal responses to stress in females compared with males [14].

Moreover, the correlation between hyperarousal and higher levels of negative and positive emotionality was found in both genders [15] but, when compared exclusively on the hyperarousal trait, women generally reported higher scores [16]. 

The construct of hyperarousal is thought to represent a specific cluster of PTSD symptoms, but it also comprises and contributes to rumination [17], sleep disturbances [18], and hyper-activation symptoms, which are more frequently observed in female patients [19]. 

Despite the increasing research interest on hyperarousal, to date there are very few measures assessing the hyperarousal trait. Potential candidate questionnaires include the H-Scale used in studies of insomnia patients [5,7]; nevertheless, data on psychometric properties of this questionnaire are still scarce [20].

The H-Scale, also known as HAS [21,22], measures information processing, tendencies to introspection, thinking about feelings, intense responses to unexpected stimuli, and other behaviors that putatively involve cortical arousal. Higher scores correspond to a heightened state of arousal; in the original publication, a total score of ≥40 had a sensitivity of 90% and specificity of 100% for identifying subjects with primary insomnia versus controls [23]. Nevertheless, Khassawneh et al. [24] found that subjects with sleep disorders obtained a mean score ≥ 29 on H-Scale, whereas controls obtained a mean score < 26. The H-Scale is assumed to assess the effect of both cognitive and somatic hyperarousal [25], but a standardized cut-off point is not available; the unavailability of instruments has even brought some researchers to use the self-report H-Scale combined with the hyperarousal items by the PTSD Checklist and the Clinician-Administered PTSD Scale (CAPS) [26].

Hyperarousal is also assessed by a subscale from the Impact of Event Scale-Revised (IES-R) [27,28], a measure of subjective distress caused by a specific traumatic event. IES-R is composed of 22 items across three subscales: intrusion, avoidance, and hyperarousal. The total score is obtained by summing the items, with higher scores indicating higher levels of traumatic response. 

Furthermore, hyperarousal can be measured in children by the Physiological Hyperarousal Scale for Children, a specific instrument developed by Laurent, Catanzaro, and Joiner [29] which does not fit the adult population.

In conclusion, the most frequently used questionnaire assessing hyperarousal levels is the H-Scale [30], which is available only in English and Swedish [16].

To our knowledge, there are no valid and reliable measures for evaluating the hyperarousal trait in the Italian population. This factor greatly limits studies and research that, as we have seen, include important clinical aspects that are transversal to various mental disorders, making it difficult to compare data and restraining the generalizability of results for the reference population. 

Hence, there is a need for valid and reliable measures for assessing the hyperarousal trait for application in evidence-based research studies. To date, there are no adaptations of the H-Scale for use in the Italian context. The present study aimed to develop and validate an Italian version of the H-Scale in a sample of nonclinical adults, with particular attention towards age and gender differences. 

## 2. Method

### 2.1. Translation, back Translation, and Cultural Adaptation of the H-Scale

The H-Scale was translated into Italian by two psychiatrists and two psychologists, all Ph.D., who were well-skilled in English. The first version of the questionnaire was back-translated into English by an English teacher. After back-translation, comparison, and modification of the no-matching items, the final version of the translated scale was formed. 

Cultural adaptation of the Italian version was accomplished among university class students. Conceptual equivalence and semantic equivalence were investigated to make a further final revision. Discrepancies emerging from this procedure were discussed until an agreement on a common version was reached.

### 2.2. Data Collection and Procedures

The three psychological instruments—in order H-Scale, the Anxiety Sensitivity Index-3 (ASI-3), and the 36-Item Short-Form Health Survey (SF-36)—were inserted in an online tool panel for data collection (i.e., Google Forms^®^) and sent as invitation to participate in the research through institutional mailing lists, posts on social network sites such as Facebook^®^ and Linked-In^®^, other professional mailing lists, and web advertising. The web page remained available for approximately two months, during Summer 2018. 

The research method avoided incomplete protocols since the online application did not allow to proceed when one response was left unanswered. The informed consent, which briefly explained the research purposes and guaranteed anonymity, has been indicated in the header of the web page. The consensus was considered valid only when respondents submitted the fully compiled interviews. Answering the items take about 20–30 min, and smartphone use was allowed. 

All procedures had been conducted according to the Declaration of Helsinki. The entire study procedure followed the International Test Commission (ITC) guidelines on quality control in scoring, test analysis, and reporting of test scores [31]. We reported how we determined all data exclusions, all manipulations, and all measures in the study [32].

### 2.3. Participants

The total study sample consisted of 982 Italian adults, 526 women (53.6%) and 456 men (46.4%) aged between 18 and 80 years (Mean = 35.61±12.47). The whole sample was divided into three subgroups according to age: 

young adults, from 18 to 30 years (N = 447; % = 45.5);

middle-aged, from 30 to 50 years (N = 395; % = 40.2); 

older adults, from 51 to 80 years (N = 140; % = 14.3). 

The educational level was a high-school diploma for 39% of the subjects (N = 386) and graduation for the remaining 61% (N = 596). The gender groups significantly differed in age (females vs males mean age (S.D.) = 36.9 (12.6) vs 34.4 (12.3); *t* = 3.216; *p* = 0.001); moreover, age groups presented significant differences in gender distribution (X2 = 18.508; *p* < 0.0001).

### 2.4. Instruments

Socio-demographic and personal information—participants completed a socio-demographic form (gender, age, and level of education).

### 2.5. Hyperarousal Scale [5]

The H-Scale consists of 26 items that assess the hyperarousal behavioral trait on a four-point Likert-type scale coded as 0 = not at all, 1 = a little, 2 = quite a bit, and 3 = extremely. 

The scale produces a Total Summation Score (HSUM); a score of “introspectiveness”, i.e., a possible tendency to ruminate, including six items (4; 5; 9; 11; 22; 23), score range 0–18; “reactivity”, i.e., the “startle response”, including three items (6; 12; 17) score range 0–9; and “extreme responses” referring to the total number of items checked as “extremely” ranging from 0 to 26. The higher the total (max. 78), the higher the level of hyperactivation.

### 2.6. Anxiety Sensitivity Index-3 (ASI-3) [33]

The ASI-3, Italian version [34], is an 18-item self-report questionnaire composed of three 6-item subscales: 

—Physical Concerns, items 3, 4, 7, 8, 12, 15 (sample item: “It scares me when my heart beats rapidly”);

—Cognitive Concerns, items 2, 5, 10, 14, 16, 18 (sample item: “When my mind goes blank, I worry there is something terribly wrong with me”);

—Social Concerns, items 1, 6, 9, 11, 13, 17 (sample item: “It scares me when I blush in front of people”).

Participants were asked to indicate the extent to which they agreed or disagreed with each item on a 5-point Likert scale ranging from 0 = very little to 4 = very much. Subscales (range = 0–24) and total (range = 0–72) scores were calculated by summing relevant items. Nonclinical subjects obtained a mean of 12.19 ± 9.22. Finally, higher scores indicated a higher level of anxiety sensitivity. In the present study, ASI-3 achieved an alpha value of 0.90, showing excellent reliability. 

### 2.7. 36-Item Short-Form Health Survey (SF-36) [35]

For the assessment of the health profile, the SF-36 was used. The scale consists of 36 items subdivided into 8 health scales, i.e., general health (GH), physical functioning (PF), role physical (RP), bodily pain (BP), vitality (VT), role emotional (RE), social function (SF), and mental health (MH). 

Items have different answer options (2, 3, 5, or 6). For several areas, furthermore, the coded score on the evaluation sheet has been recorded, or in other words, reversed, but in some cases also calibrated so lower scores would indicate a worse health-related quality. 

In the present study, the raw scores were calculated for each area by summing the scores of the individual items. If more than 50% of the items in an area were not evaluated, the score for that area was considered missing. The raw scores obtained, to be comparable with each other, were then transformed on a scale from 0 to 100. Higher scores of SF-36 indicated a higher rate of health-related quality of life. In the present study, the SF-36 obtained an alpha value of 0.78, showing good reliability. 

### 2.8. Statistical Method

The Statistical Package for the Social Sciences SPSS v. 16.0^®^ (IBM, Armonk, NY)was used for data analysis. Student’s t-test for independent samples and chi-square tests were performed to evaluate differences among groups on demographic features. To verify the psychometric properties of the translated H-Scale, Pearson’s correlation and ANCOVA with Bonferroni post-hoc tests were used; eta-squared statistic was provided to represent the effect size. The significance level for the test was *p* < 0.05; additionally, Bonferroni correction was performed for the correlation analyses.

## 3. Results

### 3.1. Descriptives

**Figure 1:**
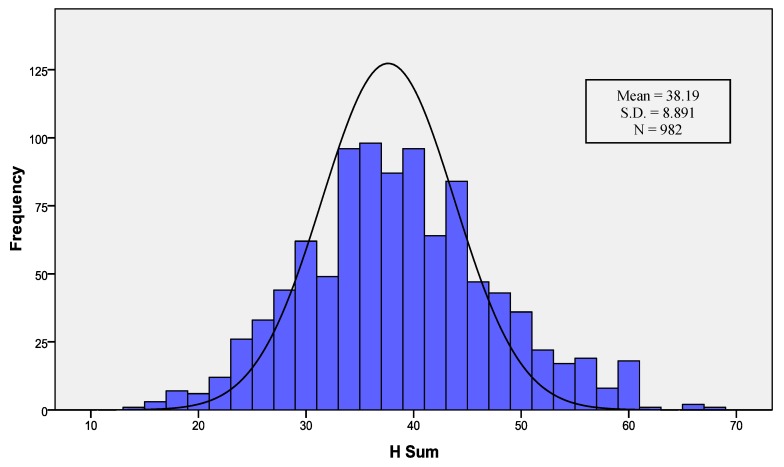
H-Scale sum distribution, histogram with a normal curve.

Table 1 shows the descriptive statistics of the whole sample. The median and the mode resulted around 38.19 (SD = 8.89) with a mode score of 40.00. Skewness and kurtosis values suggested that respondents were distributed as a normal/Gaussian curve (see Figure 1). Subjects obtained a minimum of H-sum of 14/0 and a maximum of 67/78, loading the 86% of the possible maximum score. The percentile cut-offs are also presented in Table 1.

### 3.2. Reliability and Internal Consistency

To verify the reliability of the scale, all the items were tested with the reliability analysis for the scale. The result indicated that the H-Scale internal consistency was adequate. Cronbach’s alpha coefficient was 0.81, above the recommended 0.70 threshold, and could be slightly improved by deletion of item 1 (α = 0.82). Since the reliability was in both cases very high, item 1 was preserved. 

### 3.3. Concurrent and Discriminant Validity

To evaluate the concurrent validity, Pearson’s correlation coefficient was performed. We hypothesized positive correlations with anxiety sensitivity and negative correlations with physical and mental health. As can be observed in Table 2, all aspects of anxiety sensitivity (i.e., the physical, cognitive, and social concerns) were positively correlated with all the H-Scale dimensions, such as introspectiveness, reactivity, and extreme response counts. Furthermore, there was a strong correlation even between the H-sum and the ASI-3 total score, which indicated the inter-relation between the constructs of hyperarousal and anxiety sensitivity. When the Bonferroni correction was performed for the number of hypotheses, scores did not change, and correlations between subscales remained statistically significant.

To test the discriminant validity, the relationship between hyperarousal and general health, as assessed by the SF-36, was evaluated through Pearson’s correlation coefficients. Results showed, as expected, negative correlations (all with a significance level of *p* < 0.001) among H-Scale subscales (introspectiveness, reactiveness, and extreme responses) and total score, and all SF-36 subscales. Furthermore, the absolute correlation coefficients were lower for the discriminant analyses than for the concurrent analyses. Findings indicated that the higher the H-Scale scores (hyper-activation) the lower the general health, including mental health (Table 3). 

When the Bonferroni correction was performed for the number of hypotheses, scores did not change and correlations between subscales remained statistically significant, except for “introspectiveness/physical functioning” and “extreme/change in health status” correlations. 

### 3.4. Age and Gender Differences

We performed ANCOVA (with gender as covariate) and a Bonferroni post-hoc test to compare the three groups stratified by age. Age differences are shown in Table 4. Our results suggest that scores of the H-Scale were age-dependent. In particular, the scores of both introspectiveness and reactivity, along with extreme responses and H-sum, tended to decrease with increasing age/maturity. Young adults, compared with middle-aged and older subjects, showed higher scores on “introspectiveness” (*p* < 0.0001; Bonferroni post-hoc test: young adults vs middle-aged: *p* < 0.0001; young adults vs older adults: *p* < 0.0001), “extreme response” (*p* < 0.0001; Bonferroni post-hoc test: young adults vs middle-aged: *p* = 0.003; young adults vs older adults: *p* = 0.005), and “H-sum” (*p* < 0.0001; Bonferroni post-hoc test: young adults vs middle-aged: *p* < 0.0001; young adults vs older adults: *p* < 0.0001). Furthermore, young adults scored higher than middle-aged subjects (*p* < 0.0001; Bonferroni post-hoc test: young adults vs middle-aged: *p* < 0.0001) on “reactivity” subscale. No significant differences emerged between middle-aged and older subjects. Effect sizes, according to eta-squared, were large in “introspectiveness” and “H-sum”, and medium in “reactivity” and “extreme responses” dimensions.

Furthermore, we conducted an ANCOVA (with age serving as covariate) to compare scores of the H-Scale between males and females (Table 5). Our results indicated that women showed a higher ruminative tendency as suggested by subscale “introspectiveness” (*p* < 0.0001), and an increased startle response as assessed by the items belonging to the “reactivity” subscale (*p* = 0.004). Moreover, women more easily tended to check as positive the “extreme response” (*p* < 0.0001), thus obtaining higher total scores, as compared to men (H-sum *p* < 0.0001). Effect sizes, according to eta-squared, were medium in all explored H-Scale dimensions and total score.

## 4. Conclusions

The present study was aimed to test validity, reliability, and psychometric properties of the Italian translation of the H-Scale in a nonclinical sample of Italian-speaking adults. The concurrent validity was primarily assessed by correlating the H-Scale with the ASI-3. Findings revealed positive correlations between the hyperarousal measure and the Anxiety Sensitivity Index; the strength of correlations indicates that the construct is congruent, but not overloading. Moreover, to further test discriminant and concurrent validity, H-Scale has been correlated with a dissimilar instrument, the SF-36 scale; in this case, negative correlations have been obtained. In other words, hyperarousal was negatively associated with health components: the higher the level of hyperactivity, the lower the health-related quality of life. 

In this study, H-Scale reliability has been demonstrated by additional internal consistency analyses, whose results are partially congruent with the previous Swedish validation study [16]. As regards gender differences, we found that women reported higher scores in all H-Scale subscales than men. Literature demonstrated higher levels of hyperarousal-related psychiatric disorders, such as PTSD and MD among women. According to our results, the hyperarousal trait was more prominent even in a nonclinical sample of women from the general population.

Furthermore, we found that older subjects showed lower scores on the H-scale and H-subscales. This result is in contrast with findings from the Swedish validation study, in which no evidence of age trend was documented; however, it should be noted that the Swedish study had enrolled middle-aged and aged subjects (40 years or older). It could be hypothesized that the ability to self-regulate arousal may increase over the years; however, this possible explanation cannot be addressed here, and it probably deserves further studies and/or meta-analyses. 

The Italian version of the H-Scale appears to be a reliable and valid self-report instrument for the assessment of hyperarousal behavioral traits, and it can be suitable for clinical research in the Italian population as well as for multi-country studies. Cronbach’s alpha showed an acceptable score for the internal consistency of the scale. These findings add further knowledge on the psychometric properties of this questionnaire [20].

Similarly to Buysse et al. [23], we obtained a mean that was also the median value, but a clinical cut-off is still missing. To date, H-Scale has been applied in sleep research, and main results have shown that subjects with insomnia scored significantly higher than controls with no sleep disorders [24]. To the best of our knowledge, this is the first study to investigate the Italian translation of this instrument in a nonclinical population, providing elements for the first acquisition of statistical normative data. 

This study has several limitations that should be considered. A major limitation is that, aside from the self-report scale, no objective assessments of hyperarousal have been included. Jawinski et al. [36] used an automated EEG-based algorithm to measure brain arousal in the resting state and demonstrated an association with self-reported daytime sleepiness. In the same vein, EEG results might be compared to results of the H-scale. Interestingly, in line with the findings of Szelenberger and Niemcewicz [9], this EEG-based algorithm has been applied in clinical samples with psychiatric diseases believed to have arousal-related pathophysiologies [37,38,39]. In sum, future studies may further investigate the overlap between subjective scores of the H-Scale and objective measures, both in clinical and nonclinical samples. 

Normative data have been recruited in an adequately large sample from the general population, and findings should be verified in clinical samples of patients. As previously reported, the connections between hyperarousal, insomnia, and post-traumatic experiences have been explored. Since hyperarousal symptoms may contribute to the clinical presentation of mood and stress-related disorders, it would be interesting to examine their potential role in other mental disorders, such as personality disorders, obsessive-compulsive disorder, and panic disorders. Furthermore, although there are clearly undeniable advantages of web-based assessments, such as cost reductions if compared with paper-and-pencil methods, the lower probability of receiving socially desirable responses, the immediate availability of data in electronic format, and the elimination of multiple responses, it should be borne in mind that web-based administration may oversample subjects from socially advantaged groups characterized by the availability of electronic devices and high literacy levels. 

Beyond the above-mentioned limitations, the Italian version of the H-Scale is a valid and reliable candidate to evaluate the hyperarousal behavioral trait in further research on a range of psychiatric disorders. 

## Figures and Tables

**Table 1:** Descriptives of the H-Scale in the study sample.

H-Sum		
Mean		38.19
Median		38.00
Mode		40.00
Std. Deviation		8.89
Skewness		0.237
Std. Error of Skewness		0.078
Kurtosis		0.028
Std. Error of Kurtosis		0.156
Minimum		14
Maximum		67
Percentiles	25°	33.00
	50°	38.00
	75°	44.00

**Table 2:** H-Scale correlations with ASI-3.

H-Scale	Introspectiveness	Reactivity	Extreme	H-Sum
Physical Concern	0.321 *§	0.411 *§	0.305 *§	0.428 *§
Cognitive Concern	0.470 *§	0.467 *§	0.412 *§	0.546 *§
Social Concern	0.408 *§	0.437 *§	0.314 *§	0.501 *§
ASI-3 Total	0.469 *§	0.516 *§	0.403 *§	0.578 *§

* *p* < 0.05; § Statistical significance after Bonferroni correction (α = 0.003).

**Table 3:** Correlations between H-Scale and SF-36.

SF-36	H-Scale			
	Introspectiveness	Reactivity	Extreme	H-Sum
Change in health status	−0.110*§	−0.139*§	−0.086*	−0.137*§
Physical Functioning	−0.069*	−0.151*§	−0.151*§	−0.140*§
Role Physical	−0.203*§	−0.244*§	−0.193*§	−0.266*§
Bodily Pain	−0.197*§	−0.231*§	−0.166*§	−0.253*§
Vitality	−0.393*§	−0.347*§	−0.323*§	−0.469*§
Role Emotional	−0.374*§	−0.317*§	−0.290*§	−0.432*§
Social Function	−0.438*§	−0.361*§	−0.378*§	−0.495*§
Mental Health	−0.465*§	−0.431*§	−0.392*§	−0.533*§
General Health	−0.323*§	−0.316*§	−0.268*§	−0.395*§

* *p* < 0.05; § Statistical significance after Bonferroni correction (α = 0.001).

**Table 4:** Age differences in H-Scale.

H-Scale	Young adults		Middle-aged		Older adults		ANCOVA		
	Mean	SD	Mean	SD	Mean	SD	F (2)	Sig.	η2
Introspectiveness	10.96	2.68	9.95	2.95	9.52	2.76	15.260	<0.0001	0.201
Reactivity	4.02	1.74	3.47	1.93	3.66	1.79	7.329	<0.0001	0.139
Extreme Responses	3.91	3.60	3.12	3.31	2.85	3.39	6.472	<0.0001	0.126
H-Sum	40.33	8.50	36.68	8.72	35.62	9.07	19.354	<0.0001	0.223

**Table 5:** Gender differences in H-Scale.

H-Scale	Males		Females		ANCOVA			
	Mean	SD	Mean	SD	F	df	Sig.	η2
Introspectiveness	10.08	2.82	10.58	2.88	21.631	2	<0.0001	0.088
Reactivity	3.61	1.90	3.87	1.78	5.563	2	0.004	0.069
Extreme Responses	3.17	3.68	3.69	3.28	8.911	2	<0.0001	0.074
H-Sum	37.20	8.92	39.05	8.77	24.512	2	<0.0001	0.104
